# Fox, Georgia L. The archaeology of smoking and tobacco. 169 pp., illus., bibliogr. Gainesville: Univ. Press of Florida, 2015. £64.95 (cloth)

**DOI:** 10.1111/1467-9655.12417

**Published:** 2016-04-27

**Authors:** Andrew Russell

**Affiliations:** ^1^ Durham University

This is, extraordinarily, the first study in historical archaeology that attempts an overview of tobacco and smoking‐related activities and what the archaeological record of this plant and its associated artefacts – particularly the tobacco pipe – tells us about society, economy, and consumption in the historical past. Using her own archaeological research on 21,575 white clay tobacco pipes recovered from Port Royal, Jamaica, as well as a wide variety of secondary sources, Fox takes us on what can become a somewhat giddying journey investigating the materials and meanings of tobacco and smoking across the Atlantic world. As someone trained as a social anthropologist and hence as an outsider to the precise and sometimes pernickety world of archaeological research, I found the collation of numerous research reports, theses, and edited volumes pertaining to the somewhat specialized field of tobacco pipes to be very helpful. Most of the time the focus is on North America and the Caribbean – this is, after all, a book in the ‘American Experience in Archaeological Perspective’ series – but we sometimes find ourselves exploring English pipe‐making kilns, looking at Dutch masters, or even, towards the end, in conversation with a businessman in Wenzhou, China. Fox's theoretical approach is to combine elements of world systems analysis and consumer theory in order to understand the crucial role that tobacco addiction played in both the formation of the early American republic and the development of what rapidly became a world system of colonization and trade.

This is an ambitious enterprise for a slim volume, and, given its brevity, there are places where the account verges on the superficial. While this book presents a historical archaeology, more could be and has been said about the ‘deep history’ and archaeology of precolonial Native American tobacco use than the few pages Fox is able to accord it. Likewise, there are other art forms and academic disciplines which could usefully be included in assembling a fuller picture of past smoking cultures. Poems and novels, as well as musical and dramatic works, also have much to tell us, information that is now becoming increasingly accessible with recent developments in the digital humanities. The brutalization associated with enslavement to tobacco (in one of the multiple ways that term can be understood) is chillingly alluded to in a sentence describing how African captives, many of them destined for American tobacco plantations, were sometimes given tobacco and pipes to ‘placate’ them during the Middle Passage (p. 80). Given that African slaves were no strangers to tobacco in their places of origin, and that the French introduced the supposedly traditional calumnet pipe to indigenous populations of the Lower Mississippi Valley, it is strange that William Jankowiak and Dan Bradburd's seminal 1996 *Current Anthropology* paper ‘Using drug foods to capture and enhance labor performance’ is missing from the list of references. The complex and multifaceted dangers of addictive enslavement are further euphemized by acknowledging the ‘strong presence of tobacco throughout the American experience’ (p. 134), while sidelining the trope of resistance to its beguiling charms. This dates well back into the pre‐cigarette era with which Fox is primarily concerned, as is referenced in Michael Nassany's foreword, where he discusses sites where the absence of tobacco paraphernalia is as significant as its presence elsewhere.

Conversely, readers can find themselves mired in archaeological arguments which seem unnecessarily detailed in a book of this length and nature. Are the ‘lively debates and deep disagreements’ (p. 61) between archaeologists over who made red clay pipes and why really worth four pages of elucidation, particularly when we return to the question again twenty pages later? Do we also need such detail about South's ‘Brunswick Pattern of Refuse Disposal’ (p. 114) or the relative consistency of the Harrington/Binford method of pipe‐stem dating (p. 119) in a chapter with the promised intent of exploring the capitalist world system (p. 105)? The conclusion betrays a surprising dullness of vision for someone versed in world systems analysis and consumer theory. Tobacco is described as ‘a binder of human experience regardless of gender, class, or ethnicity’ (p. 133). I wonder if the slaves who produced so much of it would agree? Smoking can indeed be used to ‘assert notions of individuality and identity … regardless of the possible outcomes, including stained and worn teeth, addiction, or lung cancer’ (p. 134), but cardiovascular disease and chronic obstructive pulmonary disease (at the very least) should be added to this list; the latter is due to become the third leading cause of death world‐wide by 2030. Such heavy tolls of morbidity and premature mortality have profound effects on families and friendship groups, not just the individual smoker. Finally, ‘modern advertising, the arts, and popular media’ have not merely ‘broadcast public sentiments about tobacco and smoking’ (p. 133), they have been actively manipulated by a transnational tobacco industry which has done everything it can to promote the ruggedly individual Marlboro Man beyond American shores.

There are also some trifling inaccuracies with the rendition of things on the English side of the Atlantic world. The college where boys were whipped during the plague year 1665 for not smoking tobacco is Eton, not Eaton (p. 54), and is located in a town of the same name, not London. Documents using pounds, shillings, and pence before decimalization of the British currency in 1971 need converting at a rate of 12 old pence to 5 new ones, making historical pipe prices 2.4 times cheaper than quoted on p. 44. These quibbles aside, this book makes a significant contribution to the historical archaeology of a plant that, for better or worse, has ‘revolutionized the world and changed the course of history’ (p. 1). As such, the book will be of value not only to archaeologists but also to anyone interested in the mutually dependent history of this loathsome weed and world capitalism.

